# Supplementary material for: The expert's knowledge combined with AI outperforms AI alone in seizure onset zone localization using resting state fMRI
Source: Front Neurol. 2024 Jan 11;14:1324461. doi: 10.3389/fneur.2023.1324461 (PMC10808636; doi:10.3389/fneur.2023.1324461)
Supplement: Supplementary file 1 [file Data_Sheet_1.docx]

1. **The deep learning techniques we implemented for *NOISE* and** $\bar{\boldsymbol{NOISE}}$**ICs classification (Table I):**

**Convolutional Neural network (CNN):** A 2D CNN was used with tuned hyperparameters. Details are mentioned in the manuscript as this deep learning technique was used for ***NOISE*** and $\bar{\boldsymbol{NOISE}}$IC classification.

**Multilayer Perceptron (MLP)**: MLP was trained with the best hyperparameters using a Keras-tuner for the ***NOISE*** and $\bar{\boldsymbol{NOISE}}$ICs.

**Transfer Learning:** We used the VGG16 pre-trained model as a feature extractor and defined our own classifier model. We used the pre-trained weights of ‘imagenet’ in Keras, we added flatten, dense and output layers after the last layer in VGG16. The dense layer’s number of neurons were tested for 5 different values: 192, 256, 512, 712 and 1024, out of which 512 gave the best accuracy. Fine-tuning the VGG model by freezing a few of its layers did not give good results.

**Problem Reduction**: MELODIC software decomposes the 4D data of fMRI into spatial and temporal components. We used the images of the BOLD signal time courses and power spectrum from rs-fMRI to train our CNN model.

**Vision Transformer:** We implemented Vision Transformer (ViT) to evaluate its performance for ***NOISE*** and $\bar{\boldsymbol{NOISE}}$IC classification. The hyperparameters were obtained using “Optuna”, and to avoid gradient explosion and gradient vanishing issues, we implemented gradient clipping and batch normalization respectively.

**Table I: Deep learning results for *NOISE* and** $\bar{\boldsymbol{NOISE}}$**ICs classifications.**

| Technique | Accuracy | Precision | Sensitivity | Specificity |
| --- | --- | --- | --- | --- |
| CNN | 80.3% | 81.8% | 76.2% | 83.4% |
| MLP | 54% | 52.4% | 60.4% | 47.8% |
| Transfer Learning | 75.5% | 76.2% | 77.8% | 73.1% |
| Problem Reduction | 74.5% | 72.6% | 75.5% | 73.5% |
| ViT | 69.01% | 69% | 71% | 67% |

As CNN reports the best classification results, we used this deep learning technique for noise elimination.

1. **Notations used in the paper and their definitions:**

**Table II: Notations used in the paper and their definitions.**

| Notation | Definition |
| --- | --- |
| $\boldsymbol{I}$ | Independent component |
| $\boldsymbol{I}_{\boldsymbol{R}}$ | Resized independent component |
| $\boldsymbol{I}^{\boldsymbol{L}}$ | Labelled independent component |
| $\boldsymbol{I}_{\boldsymbol{R}}^{\boldsymbol{L}}$ | labelled Resized independent component |
| $\boldsymbol{I}_{\boldsymbol{R}}^{\boldsymbol{L\{N\}}}$ | labelled Resized set of ***Noise*** independent component |
| $\boldsymbol{I}^{\boldsymbol{L\{R,S\}}}$ | labelled Resized set of ***RSN*** and ***SOZ*** independent component |
| $\boldsymbol{\rho}$ | Confidence score of SOZ classification given by EKI |
| $\boldsymbol{F}_{\boldsymbol{ex}}$ | Features extracted from expert knowledge |
| $\boldsymbol{\omega}_{\boldsymbol{ex}}$ | Weights determined using expert knowledge |
| $\boldsymbol{I}^{\boldsymbol{SOZ}}$ | SOZ classified independent component |

1. **Statistical pattern learning with expert knowledge (SLEK)**: The technique proposed in Hunyadi’s paper was replicated. rs-fMRI ICs and BOLD signal features were extracted from the IC images. Features extracted were number of clusters greater than 135-pixel size, activation originating from grey matter and moving towards ventricles while overlapping on the white matter, sparsity in activelet basis using the Gini index metric, and sparsity in sine basis using Gini index metric. To do a fair comparison our proposed approach, we also applied SMOTE technique on this replicated technique for the SOZ ICs features i.e., new synthetic features of SOZ ICs were generated using SMOTE. The features extracted from the rs-fMRI images and BOLD signals of ***NOISE***, ***RSN*** and ***SOZ*** were then used to train a Least Squares Support Vector Machine (LS-SVM) as described in the paper.
2. **Unsupervised learning with expert knowledge (ULEK)**: **ULEK** uses six expert rules in stage one for an IC to be classified as noise, combined from Boerwinkle and Hunyadi works. The second stage works on ***RSN*** and ***SOZ*** classification using their biomarkers.

a) Voxel cluster detection algorithm: A density-based scanning approach is undertaken to derive voxel clusters. The output of this step is the set of clusters in each IC slice.

b) Brain boundary/periphery detection: Contours in the brain are derived using a Sobel filter-based edge detection technique. The technique extract brain boundary by examining the biggest contour in the image that encompasses cerebrospinal fluid and blood vessels.

c) White matter detection: The white matter manifests as the brightest contour in the brain. The blood vessels and cerebrospinal fluid in the white matter contour are discarded.

d) Blood vessel detection: The major basal-region blood vessels present themselves as low intensity contours encompassed in the brain periphery contour.

e) ***NOISE*** IC classification: Utilizing the a, b, c, and d steps, an IC can be classified as ***NOISE***. From each slice of an IC the clusters, and contours are extracted. Next, the overlap of the largest cluster to the brain boundary, intersection of the largest cluster with the white matter, and intersection of the cluster with blood vessels is evaluated. The output of the first stage classifier is a statistic for each slice on cluster size, percentage (%) overlap with brain boundary, % overlap with blood vessels, and % overlap with white matter for each cluster in a slice. Each IC has multiple slices depending on the sampling rate of the fMRI device (55 for the shared dataset).

The second stage classifier sorts the slices in decreasing order of cluster sizes. It selects the top 10 slices and checks the percentage overlap to determine slices that are noise. If the majority of the top 10 slices are noise, the IC is classified as ***NOISE***. If the IC passes through the majority evaluation, it is passed to the second level classifier to determine if it is a normal ***RSN*** or ***SOZ***. The ***SOZ*** biomarkers such as asymmetry in voxel clusters between two hemispheres of the brain, low number of big clusters in one hemisphere, or high frequency components in the BOLD signals, as used in Hunyadi et al. paper, are extracted from each IC. Each ***SOZ*** marker contributed to the likelihood of an IC to be ***SOZ*** localizing through a Gaussian distribution with mean and standard deviation computed across the whole set of ICs. When the likelihood crossed the threshold of 0.8, the IC was classified as ***SOZ***.

1. **Statistical Analysis**

**Justification for using ttest:**

**Table III: The variance in evaluation metrics across test data subsets and the result of Kolmogorov Smirnov (KS) test on the evaluation metrics. P value of KS test evaluates the significance of whether the null hypothesis that data belongs to normal distribution was rejected.**

| **Method** | **Accuracy**  **variance** | **KS test P value** | **Precision**  **Variance** | **KS Test P value** | **Sensitivity**  **variance** | **KS Test P value** | **F1 Score**  **Variance** | **KS Test P value** |
| --- | --- | --- | --- | --- | --- | --- | --- | --- |
| SLL-CNN | 4.4 | 0.94 | 8.07 | 0.82 | 5.42 | 0.92 | 4.3 | 0.98 |
| ULEK | **4.55** | 0.92 | **4.56** | 0.45 | **7.88** | 0.77 | **2.9** | 0.76 |
| SLL-ViT | 4.7 | 0.98 | 10.6 | 0.96 | 5.7 | 0.77 | 5.3 | 0.98 |
| SLEK | 15.4 | 0.57 | 6.8 | 0.74 | 18.6 | 0.97 | 14.12 | 0.72 |
| SLLEK | 4.73 | 0.96 | 4.7 | 0.59 | 6.73 | 0.96 | 2.77 | 0.97 |

The variance of the method that is closest to our approach SLLEK is shown in bold face in Table III. The table shows that the variances of SLLEK and the closest technique ULEK were less than 10% apart. We consider that as similar variance. We performed the Kolmogorov-Smirnov test to determine goodness of fit for the evaluation metrics to a normal distribution. A p value < 0.05 in Table III indicates that the evaluation metrics do not belong to a normal distribution. Since all p values were higher than 0.05, we conclude that the evaluation metrics follow a normal distribution. The similar variance of the two compared techniques and the normal distribution of the evaluation metrics meet the conditions for using one sided ttest to evaluate the statistical significance of difference in mean values of the evaluation metrics across the compared techniques.
